# The Role of DNA Degradation in the Estimation of Post-Mortem Interval: A Systematic Review of the Current Literature

**DOI:** 10.3390/ijms21103540

**Published:** 2020-05-17

**Authors:** Pamela Tozzo, Salvatore Scrivano, Matteo Sanavio, Luciana Caenazzo

**Affiliations:** 1 Department of Molecular Medicine, Laboratory of Forensic Genetics, University of Padova, 35122 Padova, Italy; luciana.caenazzo@unipd.it; 2 Department of Cardiac, Thoracic, Vascular Sciences and Public Health, University of Padova, 35122 Padova, Italy; salvoscrivano@gmail.com (S.S.); matteored90@gmail.com (M.S.)

**Keywords:** post-mortem interval, forensic genetics, molecular biology, DNA

## Abstract

The determination of the post-mortal interval (PMI) is an extremely discussed topic in the literature and of deep forensic interest, for which various types of methods have been proposed. The aim of the manuscript is to provide a review of the studies on the post-mortem DNA degradation used for estimating PMI. This review has been performed following the Preferred Reporting Items for Systematic Reviews and Meta-Analyses and the PRISMA Guidelines. Several analytical techniques have been proposed to analyse the post-mortem DNA degradation in order to use it to estimate the PMI. Studies focused mainly on animal models and on particular tissues. The results have been mixed: while on the one hand literature data in this field have confirmed that in the post-mortem several degradation processes involve nucleic acids, on the other hand some fundamental aspects are still little explored: the influence of ante and post-mortem factors on DNA degradation, the feasibility and applicability of a multiparametric mathematical model that takes into account DNA degradation and the definition of one or more target organs in order to standardize the results on human cases under standard conditions.

## 1. Introduction

Estimating the time of death, i.e., the interval between death and the examination of the deceased person, is an important task in daily forensic caseworks, and its determination requires the calculation of measurable data along a time-dependent curve back to the starting point [1].

Over the years, various techniques have been developed to estimate the time of death:

- Physical processes, i.e., body cooling, post-mortem lividity and radiocarbon dating;
- Physico-chemical processes, such as rigor mortis and supravital reagibility of skeletal muscle;
- Bacterial processes, i.e., putrefaction;
- Chemical methods, based on metabolic processes, autolysis and diffusion;
- Entomological approaches.

Of the methods published, the majority can be divided into two main categories: those occurring within the early post-mortem period and those during the late post-mortem period [2].

During the early post-mortem period (few hours since death), supravital reactions—defined as post-mortem excitation-induced reactions of tissues—can be examined. Immediately following the early signs of death, the later post-mortem changes develop, which cannot strictly be separated from the early ones. Later post-mortem changes do not only lead to the decomposition of the corpse, but in some cases also to its preservation. Endogenous processes resulting in decomposition are autolysis, putrefaction and decay while exogenous factors include animal predation, exposure to the elements and mechanical injury.

*Livor mortis*, *rigor mortis*, and, to a lesser degree, *algor mortis* are frequently used to estimate the post-mortem interval (PMI), for the simplicity and immediacy of their use [3,4].

Other methods have been developed to define the PMI with reasonable accuracy, like bacterial (putrefaction) and bio-chemical methods (pH of blood, potassium in humour vitreous and metabolite profiles) [5,6]. These methods still involve great inaccuracies and limitations in their applications and they are dependent on environmental and geographical conditions and individual characteristics (age, sex, and physiological and pathological states of the deceased).

Recently, a large number of experimental approaches have been explored to accurately determine the time of death. A precise evaluation of PMI, in fact, would require a parameter that changes constantly and proportionally from the time of death with a linear process [7]. 

The DNA molecule has been considered as a possible parameter for the PMI estimation, considering that it is one of the most stable components of cells, its content is similar among different individuals and different cell types within the same species and its denaturation, in biological samples, begins immediately post-mortem (caused by endogenous nuclease activity and hydrolytic attack) and continues at a constant rate, regardless of the temperature and the mechanisms of death [8,9].

During the past few decades, different methods have been published in scientific literature in evaluating the use of DNA to PMI detection [10]. Both qualitative and quantitative estimation of nuclear DNA fragmentation have been studied: from basic UV spectrometry, through dye staining and image analysis technique (IAT), to single-cell gel electrophoresis (SCGE), known as the “comet assay”, and, in the last few decades, DNA amplification methods, such as Real-Time quantitative Polymerase Chain Reaction (RT-qPCR) [11,12].

Although since early 2000′s some reviews have been published on the use of DNA for the determination of the PMI, each of them has been focused on a precise analysis methodology. In particular, Wang et al. [13] in 2002, briefly reviewed the studies concerning the principium of quantifying the DNA content by IAT and the foreground of this method for PMI estimation in forensic pathology. Five years later, Hao et al. [14] highlighted the development, the advantage/disadvantage and the potential future applications of various forensic DNA technologies. In 2011, Lin et al. [15] evaluated the studies concerning the DNA quantitative techniques used for estimating PMI, such as SCGE, Feulgen-staining image analysis and flow cytometry. 

However, until now, there has not been a comprehensive review of the studies published worldwide about all the forensic DNA quantitative techniques used for estimating PMI, with special reference to the recent advances in RT-qPCR. For this reason, with this review, we want to provide an overview of the most important scientific studies, focusing on the reliability of DNA quantitative techniques for PMI estimation, the limitations of these techniques and their possible future applications. This review has been performed following the Preferred Reporting Items for Systematic Reviews and Meta-Analyses and the PRISMA Guidelines [16].

The aim of this review is not to provide an overview of all the proposed methods for estimating the post-mortal interval, but only those related to DNA analysis. In this review, the term “post-mortem interval—PMI” will be used with the meaning of the interval between death and the examination of the deceased person.

## 2. Results

From 1988 to 2019, several studies have investigated a possible correlation between the degradation rates of nuclear DNA and PMI. These studies are summarized, in chronological order, in Table 1, in which we highlighted number, species and types of samples, temperature conditions, time frame assessed, detection methods, statistical models (if given) and remarks.

In order to make the results obtained in the current review more comprehensible to the reader, we decided to divide the manuscripts on the basis of the method used in assessing DNA degradation: by Restriction Fragment Length Polymorphism (RFLP), by DNA flow cytometry analysis, by IAT, by SCGE, by RT-qPCR analysis and by other techniques based on DNA analysis.

### 2.1. Assessing DNA Degradation by RFLP

RFLP-DNA analysis (based on Southern blotting and radioactive probe detection) relied on variations of DNA fragment lengths (0.5 to 33 kilo base pairs (kb)) generated by restriction enzymes, which cut the DNA molecule at specific sites, called “restriction sites” [50]. However, nowadays, this approach has only a historical value, but it must be considered because, although already available and already used for other purposes, it was the first method to be used for the estimation of the PMI.

The first study on this aspect was published in 1988 by Bar et al. [51]. They investigated the post-mortem stability of DNA in different human tissue samples (brain cortex, lymphatic nodes, liver, spleen, skeletal muscle, kidney, thyroid gland and blood) from 23 corpses of known PMI (6 h–19 d). They noted that the amount of degraded DNA increased proportionally to the length of the post-mortem period. They also found a good DNA stability in brain cortex, lymph nodes, skeletal muscle, and, partially, in blood, while complete DNA degradation was noted for liver in a post-mortem period of 24–36 h. In the cases involving older post-mortem samples, the DNA degradation resulted in the gradual disappearance of the longer fragments in the 15–20 kb range and thus reduced the potential evidentiary value of older samples.

Thanks to these results, researchers were interested in the potential use of RFLP-DNA analysis to assess time since death. In this regard, Perry et al. [51] evaluated DNA degradation in seven human rib bone samples from multiple individuals at specific times during incubation (up to 84 d). They highlighted that the variation of DNA degradation rate was more significant between samples exposed to different post-mortem intervals and temperatures, in different individuals. 

This work had obvious limitations: it did not evaluate, in the DNA degradation process, the role of bacterial nucleases and the influence of environmental factors such as, for example, rain and sunlight. This last study remains in the published literature the only one that uses the RFLP-DNA analysis for the estimation of PMI because, with the advances in molecular biology techniques, other more sensitive and accurate assays, which have supplanted the previous obsolete method, have been developed.

Therefore, this type of study has served to confirm the obvious relationship of the degradation of the DNA molecule with the PMI. These studies maintain a historical value, as they refer to techniques no longer used and outdated by modern DNA analysis techniques.

### 2.2. Assessing DNA Degradation by DNA Flow Cytometry Analysis

Flow cytometry is a useful method to determine the nuclear DNA content within each cell of a tissue. In this method, a sample containing moving cells, whose nucleus has been previously stained with propidium iodide, is suspended in a fluid and injected into the flow cytometer instrument, where a laser beam hits one cell at a time and, in turn, a fluorescent light is emitted. The signal is captured and converted into a graphic representation of the intensity of the fluorescence emitted that is proportional to the amount of propidium iodide bound: nuclei that have lost DNA will stain less intensely than those with the diploid amount of DNA.

In 1994, Cina et al. [3], utilizing flow cytometry for the first time, investigated the increase of the ratio of “number of cells with fragmented DNA versus number with intact DNA” as a method for estimating the PMI and, thus, the time of death. They investigated ten human spleen samples, obtained from autopsies, with known time of death, and allowed to decompose at room temperature for 2 weeks. The results suggested that nuclear DNA was degraded in a relatively predictable way over time, even if the choice to use splenic tissue can be criticized for various factors, such as the difficulty in obtaining splenic tissue, especially from decomposed or burned bodies, the variability of splenic decomposition and the tendency of the spleen to colliquate rapidly.

In 1998, Di Nunno et al. [4] tried to confirm the reliability of spleen DNA denaturation in autopsy practice. For this purpose, they obtained spleen tissue samples from 35 autopsies and left them in cold storage at 2 °C, taking samples every 12 h until 144 h after death. They found a constant relationship between the time of death and DNA denaturation, particularly within the first 72 h after death.

In a subsequent publication in 2002, the same group of researchers [21] compared data on DNA degradation of splenic tissue with those from the liver and peripheral blood (the samples were collected from 25 randomly selected autopsies in which the cause and time of death were known—24 to 65 h) to determine which cells could be sampled and preserved most easily during scene investigations for subsequent cytofluorometric analysis to aid in establishing the time of death. They concluded that hepatocyte DNA degradation was better correlated with PMI than that of spleen and peripheral blood.

This last result was confirmed by Liu et al. [24], who considered different tissue samples (heart, liver, spleen and kidney) to evaluate, with cytofluorometric analysis, the law of variation of DNA content at different PMI. Therefore, they demonstrated a descendent trend of the amount of DNA of all the viscera after death, especially in spleen tissue.

In order to overcome the limits imposed by flow cytometry analysis on spleen tissue, some research groups have started to study dental pulp. In 2003 Boy et al. [7], analysed, for the first time, the dental pulp tissue in flow cytometry evaluation of PMI, because the pulp cavity and its cellular content are protected from bacterial invasion and decomposition by the surrounding dental hard tissue [52]. They collected 57 third molars from patients 15 to 30 years of age and pulp tissue was removed at 24, 48, 72, 96, 120 and 144 h post-extraction. No constant correlation was found between time and DNA degradation during the first 144 h post-extraction, so they concluded that pulp tissue was not useful in determining the early PMI (EPMI), but it could be a good substrate in the later stages.

Instead, Long et al. [25] found, with the same technique, a significant correlation between the average DNA content and PMI in human cost cartilage and dental pulp cells, stored at different ambient temperatures (30–35 °C, 15–20 °C) for a longer time (up to 15 days) than that of Boy’s study [7]. Therefore, they demonstrated the reliability of pulp tissue for the estimation of later PMI. They also observed that there was a plateau period of 0–4 days for dental pulp cells at 15–20 °C.

A more specific and sensitive flow cytometry assay in association with a statistical analysis, was recently applied by Williams et al. [10] in order to determine the effects of temperature on cellular degradation rate because the temperature influences every metabolic and autolytic processes [53]. For this study, they analysed human spleen and brain tissues from 15 corpses left over a 96-h period of time at two temperature conditions (21 °C and 4 °C), to mimic summer and winter weather, respectively. In this way, they showed that DNA degradation was slower in brain tissues than in spleen ones and that, after 48 h, DNA fragmentation was greater in room-temperature samples than in refrigerated ones. Therefore, from this moment, brain tissue has been considered an alternate and suitable organ that does not decay as rapidly as spleen, although it cannot be used in cases of direct head trauma or drowning. 

Although the investigators found a good correlation between time since death and DNA degradation with flow cytometry, they also highlighted some limitations, such as, the difficulty in the forensic analysis of human samples without extensive manipulation and in differentiating their DNA from bacterial or fungal DNA. Hence, the existence of limits in the studies cited, together with the development of new and more sophisticated DNA analysis techniques, have made this technique unpromising for the objective of estimating the PMI.

### 2.3. Assessing DNA Degradation by Image Analysis Technique

IAT combines computer technology, optics, mathematics and morphology and is often used to obtain quantitative data from tissue samples, using analysis software that segments pixels in a digital image based on features such as colour (i.e., RGB), density, or texture. An important improvement to microscopy and digital imaging has been the development of microscopes that are used to convert stained tissue on glass slides into digital images [54]. The amount and density of the same light absorbed by different cells or tissues is variable and so-called grey diversity and it can be analysed to evaluate the relative amount of contents of the tested substances. The content of the DNA was quantitatively determined by measuring the index of distortion (ID), integral optical density (IOD), average optical density (AOD) and average grey (AG) [8].

In 2000, Lin et al. [18] used Feulgen staining and auto-TV image analysis for the first time, to estimate the relationship between the DNA content of liver cells, collected from 15 rats within the first 24 h after death, and the EPMI, concluding that the DNA degeneration rate of rat liver cells had a linear relationship to EPMI.

Several Authors have evaluated the correlation between DNA and PMI with this technique on various tissues of animal models, in particular rats, with reference to both early PMI and late PMI. The studies on this topic were those of Liu and Lin et al. [19,55,56], Chen et al. [20], Luo et al. [33] and Liu CQ et al. [23]. Among the studies published in this line of research, in 2005 Chen et al. [26] evaluated the relationship between DNA degradation and PMI in various tissue samples (heart, liver, spleen and kidney) obtained from one cadaver at 6, 12, 24 and 48 h after death, by using simultaneously, for the first time, IAT and flow cytometry. The results of this study showed a more rapid DNA degradation rate in human heart, liver and kidney in the first 6 h after death than in the spleen, which displayed the better relationship. In 2007, Chen et al. [36] used rat retinal nuclear DNA in order to investigate the relationship between retinal nuclear DNA degradation and PMI. For this purpose, they randomly divided 90 rats into 15 groups, at different time intervals from 0 to 28 h after death, and, by using Feulgen-Vans staining and IAT, they measured and statistically analysed some parameters (ID, integral absorbance (IA) and average absorbance (AA)). Therefore, they concluded that, in rat retinal nucleus, AA and IA gradually declined with the prolongation of PMI, while ID had a tendency to increase.

Since 2005, researchers have also been studying the correlation between DNA and PMI by IAT in human cadavers. Hence, Ren et al. [27] studied the changes of DNA content in 18 livers from cadavers with known PMI, sampled and smeared every hour from 4 to 36 h after death. They found that IOD and AOD declined regularly with the prolongation of PMI in 36 h and that there was a correlation between DNA content and PMI.

Similar results were obtained by Liu et al. [8], He et al. [28] and Shu et al. [29] that, evaluating the DNA degradation of human spleen and brains for up to 36 h after death, demonstrated a significant correlation between the variability of all parameters (ID, IOD, AOD and AG) and early PMI. Therefore, these parameters could be effective quantitative indices for the determination of early PMI, especially AG. 

Instead, Ren et al. [35] used, as a reference sample, human thyroid follicular epithelial cells to demonstrate a significant decrease of the average DNA content with increased PMI.

Finally, Li et al. [46] performed their work on liver tissue samples, collected from 13 corpses with known PMI (13–34 h) and smeared every hour at 10 °C, 20 °C and 30 °C, respectively. They statistically measured and analysed all the parameters (ID and AOD) for DNA degradation, showing their strong correlation with the corresponding PMI.

In conclusion, the spleen tissue was mainly used as a reference sample for the estimation of early PMI because of its reliability and rapid putrefaction. However, the IAT lacks specificity because it is unable to differentiate between eukaryotic and prokaryotic DNA mixtures. This is highly relevant for forensic samples and for the time-since-death application because it requires the use of intact tissue not exposed to bacteria, making most of post-mortem forensic samples unsuitable for this kind of analysis. Moreover, these studies had some limitations, in particular a lot of factors (such as death mechanism, environmental temperature, etc.) had to be considered in order to ensure a better consistency and accuracy of the method.

### 2.4. Assessing DNA Degradation by Single Cell Gel Electrophoresis

Single Cell Gel Electrophoresis (SCGE), also known as the “comet assay”, relies on migration of degraded DNA from cells encapsulated in agarose to determine DNA fragmentation. In this technique, a tissue sample suspension is placed on normal agarose gel pre-coated microscope slide and its DNA is completely denatured. The DNA is electrophoresed through the encapsulating agarose and samples with degraded DNA generate an image as comets, with a head region containing intact DNA and a tail containing fragmented DNA. The parameters used to assess DNA fragmentation are tail length (µm), influenced by the size of the DNA fragments, tail DNA (%) and tail moment, which is the product of the fraction of DNA in the tail [9].

For the first time, Johnson and Ferris [22] introduced the SCGE technique for PMI estimation. They applied this technique in two different models: (1) a human leukocyte model, composed of one blood sample collected after 2 h (as an indication of early PMI) and a second sample collected after 22 h (late PMI), and (2) a porcine animal model (samples taken from skeletal muscle, heart, liver and kidney and maintained at a storage temperature of 15 °C) in the early (0–72 h) PMI. They showed an increase of DNA fragmentation from 2 to 22 h after removal of blood sample from the corpse and, similarly, an increase of DNA fragmentation from 3 to 56 h post-mortem in the porcine model. The fragmentation, obtained from both models, was evident through comet assay parameters-namely, comet-tail-length and comet-tail-moment. Both the parameters revealed an approximate linear relationship with the prolongation of PMI, although comet-tail-length provided a stronger correlation than comet-tail-moment.

Similar findings were also observed by He et al. [30,31] in two different groups: rat spleen and liver cells within 30 and 72 h after death, respectively; they reported that the rate of DNA degradation had a linear relationship with an increased PMI (0–15 h and 0–18 h, respectively) and that tail-length and tail-moment showed strong statistical correlation with PMI.

Instead, Zhen et al. [34] studied eight parameters including the tail length, the head radius, the percentage of head DNA, the percentage of tail DNA, the tail moment, the olive moment, the head area and the tail area to verify their changes in DNA degradation in myocardium cells of 111 rats at different PMI up to 72 h. They observed that all these 8 parameters were greatly associated with the extension of PMI, demonstrating a close relationship of DNA degradation rate with PMI.

El-Harouny et al. [37] carried out their work on 40 rats killed by drowning and classified into 5 groups up to 24-h post-mortem. The results demonstrated that DNA degradation, as evident through comet assay parameters (olive-tail moment and tail-length) had a good relationship with early PMI in the studied organs (lung, liver, spleen skeletal muscle and brain), although it was more prominent in the lungs (beginning from 3 h post-mortem) and in the spleen (beginning from 6 h post-mortem) than in the brain.

Similarly, Gooma et al. [47] discovered, in another murine experimental model (brain and liver collected from 36 adult rats divided into 6 groups for up to 24 h post-mortem), that tail DNA, tail length, tail moment and Olive Tail Moment (OTM) increased in brain and liver tissues with increasing PMI, showing a strong correlation with EPMI (0–24 h). 

Instead, Hu et al. [38] evaluated the difference in DNA degradation in murine bone marrows and brains up to 40 h post-mortem, at two different temperature conditions (1 °C and 20 °C, respectively). They observed a different decline degree in the percentage of head DNA between brain and bone marrow cells after death (brain > bone marrow at 20 °C) and that the percentage of head DNA was more valuable for PMI estimation than tail-length and tail-moment.

Furthermore, Fang et al. [39] reported that the degree of DNA degradation of porcine retinal cells, from 2 to 24-h post-mortem, increased with the prolongation of PMI and that the percentage of head DNA showed strong correlations with PMI.

In contrast, Zheng et al. [9,40] studied DNA fragmentation in mouse skeletal muscle, heart, liver, kidney, brain and dental pulp cells using SCGE at prolonged PMI up to 72 h. These studies revealed that the parameters of nuclear DNA degradation, among all the tissues considered, were strongly correlated with the prolongation of PMI.

Finally, Zaki et al. [48] evaluated the relationship of the extent of DNA degradation and certain components of the oxidant/anti-oxidant balance in brain and skeletal muscle tissues of 40 adult rats with the PMI up to 96-h after death. They found that the histopathological alterations in brain and skeletal muscle, based on oxidant/anti-oxidant balance in favour of the anti-oxidant at 96 h, were correlated with DNA fragmentation. Therefore, there was a good correlation between increase in oxidant, DNA fragmentation and decreased anti-oxidant levels in brain and muscle tissue and PMI within 96-h post-mortem.

The findings obtained with this group of papers suggested that SCGE is a quick, but relatively insensitive assay (requiring microgram of DNA for analysis) that could be used as an adjunctive method for PMI estimation [15]. However, SCGE lacks specificity and presents the same limits of the above described techniques, being unable to differentiate between eukaryotic and prokaryotic DNA mixtures, and exhibits excessive inter-laboratory variability due to a lack of consistent protocols and analysis technique. For these reasons, SCGE has not gained the same general acceptance as PCR-based methods have gained.

### 2.5. Assessing DNA Degradation by DNA Amplification Analysis

RT-qPCR is an amplification process that allows the quantification of the number of specific DNA sequences’ copies [57]. Over the last ten years, RT-qPCR based methods have become the standard method for forensic DNA quantification. Despite this, until 2010, little or no work has been performed applying this approach to the direct assessment of DNA degradation to determine PMI.

Larkin et al. [41] used PCR method to evaluate the effects of Accumulated Degree-Days (ADD) on the DNA amount in skeletal muscle samples, taken from two pig carcasses placed above ground during the first week following death and subsequently every 10 days, in summer and winter season, respectively. ADD are heat-energy units that represent the accumulation of thermal energy that is needed for chemical and biological reactions to take place in soft tissue during decomposition; for this reason, ADD represents chronological time and temperature combined. They did not find a consistent relationship between ADD and DNA amounts in winter season, while the DNA amount decreased regularly in summer up to 64 ADD. A comparative seasonal analysis showed an overall decrease in DNA amount from 0 to 101 ADD in summer and up to 138 ADD in winter. Nazir et al. [44] reported similar findings in an experimental rabbit model of a large number of samples (60 rabbit carcasses). In fact, they observed that multiplex PCR profiles were generated until 112 ADD (7 days) from whole carcasses and body fragments, while after the seventh day no soft tissues remained in either fragmented or whole bodies. The data presented were obtained at room temperature, so it was not clear whether DNA persistence could be present for similar ADD in places with warmer temperatures.

Instead, Alaeddini et al. [43] assessed DNA degradation in human rib bone samples, taken from 12 male corpses, with a semi-quantitative multiplex PCR-based method. Every piece of bone was split into two parts and stored in two different environmental conditions (room temperature and underground burial) from 103 to 445 days after death. The obtained results showed that human mitochondrial DNA survived for longer periods in samples stored at room temperature than in burial conditions, although in their work there was no correlation between the level of DNA degradation and PMI.

Itani et al. [45] provided the first quantitative analysis of the post-mortem progress of DNA degradation, by using RT-qPCR, for estimating time of death. They examined brain, kidneys, liver and skeletal muscle samples, taken from adult rats up to the sixth week after death and left into separate airtight container at 20 °C or 4 °C. They found that the quantity of amplified DNA in the liver, kidney and skeletal muscle decreased to below the value of 10 RFU (Relative Fluorescence Units) in 1–3 weeks at 20 °C, while it decreased almost linearly for 6 weeks in the brains left at 20 °C, but not beyond the value of 20 RFU. Instead, in the dead body left at 4 °C, no decrease in brain DNA was observed, while in the liver, kidney and skeletal muscle the quantity of DNA remained above the value of 10 RFU until the end of the experiment. The results of this study demonstrated the usefulness of the brain as a sample for DNA analysis of decaying corpses and of quantifying the amplified DNA in the brain at 20 °C and in the liver at 4 °C for diagnosing time of death. They also suggested that the ratio of the quantity of amplified DNA in the liver with respect to the brain at 4 °C might be useful for the same purpose.

Ebuehi et al. [11] evaluated how post-mortem time (up to 48 h) affected the integrity of DNA extracted from the brain, liver, heart and kidney of 20 male albino rats and, then, detected by Random Amplification of Polymorphic DNA-Polymerase Chain Reaction (RAPD-PCR) followed by agarose electrophoresis. The results of the post-mortem DNA profile showed a gradual degradation of nuclear DNA in the brain, kidney and heart, with increasing PMI. In addition, they observed that the brain DNA degradation occurred at a slower rate than with the other organs and, therefore, the brain could be used as a valuable organ for studying degradation in longer PMI.

In 2019, Mansour et al. [49] collected 95 teeth from 39 corpses, which were subjected to different post-mortem conditions in real forensic caseworks to isolate DNA and evaluate DNA quantification. In comparison with previous relevant studies based on controlled environmental conditions, this investigation, based on real forensic cases, showed some consistencies as well as some inconsistencies in its results. Ante-mortem factors (sex, age, tooth type, and tooth portion) displayed no significant relations to dental DNA yield, in contrast to post-mortem factors (PMI, post-mortem condition, and the surrounding environment) that did show such relations. The highest DNA amount was observed in dental samples representing the shortest PMI and DNA concentration dropped substantially after 10 days after death. The early period after death was shown to be the most critical period with respect to yielding dental DNA. DNA isolated from dental samples older than 10 days was vulnerable to considerable damage. In this study PMI was confounded with other ante- and post-mortem factors and, moreover, its role may considerably interact with other factors such as post-mortem conditions. This study nonetheless demonstrated that high DNA concentrations were obtained from burnt and fresh corpses, whose PMI did not exceed 1–2 days.

In conclusion, these studies found that RT-qPCR is a relatively new method to estimate PMI that, despite the few studies published, seems to be more accurate and sensitive in the determination of DNA degradation as a means of PMI estimation, because it is able to distinguish between prokaryotic and eukariotyc DNA from a mixture.

### 2.6. Assessing DNA Degradation by Other “DNA Techniques”

Zhan et al. [32] used Terminal Deoxynucleotidyl Transferase (TDT) as a method of estimating PMI. TDT is a template-free polymerase that can catalyze the random addition of deoxy-ribonucleoside triphosphates (dNTP) to the 3′-OH terminus of DNA, making it widely used as a tool for the detection of target DNA using labeled oligo-nucleotides; this results in a relatively time-consuming and expensive procedure. Zhan et al. [32] detected the Deoxyuridine Triphosphate (dUTP) residues after experimental reaction as an indicator of quantity of DNA degradation, showing a decreasing tendency of the residues of dUTP with the prolongation of PMI.

Instead, Xiong et al. [42] evaluated the relationship between DNA degradation in cells of kidney and liver tissue and PMI during 48–72 h after death at 25 °C. The DNA content in different tissue cells was examined by means of confocal Raman micro spectroscopy with an excitation wavelength of 532 nm. Results show that the relative peak intensities of confocal Raman microscopy for the tissue cells decreased gradually with the prolongation of postmortem interval from 48 to 72 h after death, while the peak intensity at 1094 cm (−1) was reduced obviously; and the peak intensity at 1454 and 2923 cm (−1) did not change significantly. Authors concluded for a linear relationship between the degradation rate of DNA in the tissue cells and PMI.

## 3. Discussion

In death investigation, accurate PMI detection is a crucial issue. The techniques currently utilized for estimating PMI can be broken down into two different methods: concurrence-based and rate-of-change methodologies. The first of these methods relates or compares the occurrence of a known event, which took place at a known time, with the death, which took place at an unknown time. 

The second type of techniques measures some aspects of evidence, directly associated with the body, which changes at a known or predictable rate and is started or stopped at time of death [12]. 

The observational changes (*algor*, *livor* and *rigor mortis*) that occur in the human body after death belong to this second technique and represent the easiest way of estimating early PMI [58]. However, these post-mortem changes are unable to provide an accurate estimation of PMI because they are influenced by internal and external factors [59].

**Figure 1:**
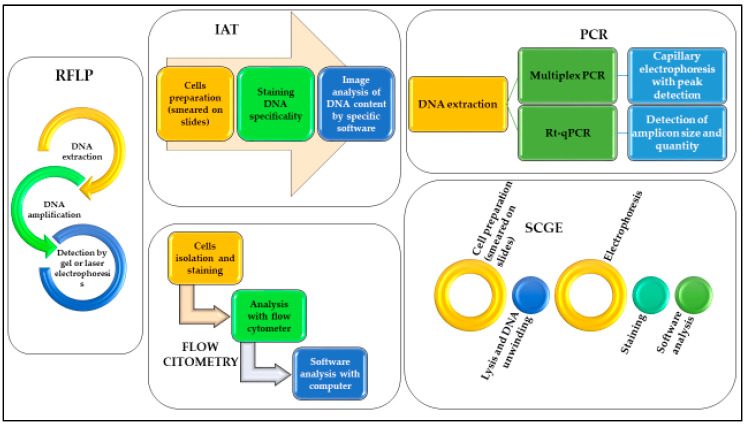
Schematic representation of the workflow necessary to use DNA analyses for the different methods described.

Since Sir Alec Jeffreys first demonstrated the potential use of DNA analysis for forensic purposes in 1985 [60], DNA analysis has revolutionized forensic science. With the advances in forensic biology techniques over the years, various and more sensitive methods have also been developed to assess whether DNA post-mortem degradation could be used for the estimation of PMI. A summary of pros and cons comparing the techniques reported in this review is provided in Table 2. In Figure 1 we report the schematic workflow of each technique.

The first researchers [17] that used RFLP technique demonstrated a variation in the rate of DNA degradation between individuals that was not as significant as between samples exposed to different PMIs and temperatures. However, the results of this work were of limited value because of the low sensitivity of the method and the failure to take into account the role of bacterial nucleases and the influence of environmental factors in the rate of DNA degradation. Hence, this method was soon replaced by other more accurate and sensitive ones, but its fundamental value is undisputed, because it was the first study in this field of research.

Flow cytometry was used, as a simple and relatively inexpensive assay, by some researchers [3,4,7,10,21,24,25] to evaluate the correlation between DNA degradation and PMI in different tissues. Several organs and tissues have been analysed, with the aim of finding the most suitable for this type of analysis. Splenic cells (analysed in mice) were recommended by several investigators [3,4,10] as a source sample to determine post-mortem period up to 72 h. Other tissues that have been evaluated included peripheral blood and hepatic tissue [21,24], the latter showing a better correlation between DNA degradation and PMI than that of spleen.

On the other hand, “non-organ” tissues suggested as suitable samples for flow cytometry analysis of PMI included cost cartilage and dental pulp [7,25], with the latter demonstrating a correlation between DNA degradation and PMI and, therefore, its reliability for the estimation of later PMI. Brain samples [10] also revealed the brain to be a suitable organ, as compared with the spleen, for estimating PMI, especially for later PMI. 

Similar findings were also observed by several investigators [8,18,19,20,22,23,26,27,28,29,30,31,33,34,35,36,37,38,39,40,46,47,48,55,56,57] that, by applying IAT and SCGE to a variety of different tissue samples (liver, spleen, kidney, skeletal muscle, marrow bone, cornea/retina, heart and thyroid), reported a linear relationship between DNA degradation and PMI-in particular, in brain, spleen and bone marrow tissue for the estimation of later PMI [20,33,37,38,56] and in the other organs for early PMI [8,18,19,22,23,26,27,28,29,30,31,34,35,36,46,47,55]. Moreover, some of these studies [28,38,46] also evaluated the effects of temperature on the rate of DNA degradation. 

In general, liver tissues were the most used source of DNA in post-mortem studies, followed by spleen and brain tissues. Such variability appears to be related to the ante-mortem ribonuclease activity of the tissue: the ribonuclease-poor tissues, such as the brain and retina, exhibit a greater nucleic acid stability than the ribonuclease-rich tissues, such as the liver and spleen [57].

However, despite the promising results achieved, flow cytometry, IAT and SCGE have a low specificity because they are unable to differentiate between intact human DNA (target sequence DNA) and non-target DNA (bacterial or fungal). Therefore, these methods have a limited value in real-world application measurements and have not gained a general acceptance in forensic community.

In the last few years, with the introduction of RT-qPCR based methods and the introduction of capillary electrophoresis assay, researchers [11,41,43,44,45,49] have started to verify whether this relatively new method could be applied for the assessment of DNA degradation to determine PMI. 

The advent and application of RT-qPCR has turned out to be a more sensitive and specific method than the previous ones. However, as few studies have been developed on this topic until now, the current results have not been concordant [43,45] on the correlation between human DNA degradation and PMI. A dependent-tissue DNA degradation rate with the prolongation of time after death has also been observed. In particular, brain tissue shows a slower degradation rate than the internal organs. Finally, it has also been suggested that the evaluation of DNA degradation was best modelled as dependent on ADD [41,44] rather than just time. However, this method differs from the others applied in this field and, given that this technique is also spreading for non-traditional applications, further in-depth studies would be necessary to verify the effective applicability to the PMI estimate.

It is currently still too early to establish whether this approach will actually be an effective adjunct method to accurately estimate PMI.

In addition, only more recently, the contribution of statistical analysis in many of the above cited studies [8,10,36,37,39,40,41,43,44,45,46,48] which proved to be essential for this field of research as well as for the determination of the PMI-based-on-RNA analysis, has allowed for the establishing of a linear correlation between DNA degradation and PMI. However, in contrast to the multi-parametric mathematical model developed by various Chinese forensic scholars [61,62], none of the methods proposed by the researchers, based on DNA degradation, have been able to assess an estimated error with the known PMI. In fact, the multi-parametric mathematical model applied to RNA analysis, was able to provide the error rate between the known and the estimated PMI, with a lower average rate in the murine experimental models than real human cases, even if not statistically significant. This mathematical model was also applied considering different temperature conditions and seemed not to be influenced by other parameters; nevertheless, it is still suffering from some limitations, such as different tissue samples and a relatively short time after death [63]. Therefore, the methods based on DNA degradation are not yet at the same level of accuracy as those based on a multi-marker RNA approach, although the PCR method’s full potential still has to be assessed.

Some studies have value because they have further confirmed known and relatively simple scientific correlations, such as that relating to the degradation of DNA in human tissues after death. Other studies are credited with having tried to correlate the relationship between post-mortem DNA degradation and time in a more stringent and useful way for medico-legal purposes. However, all these studies, regardless of the methodology used, have led to results that do not appear to be as promising as those conducted on mRNA [63]. It is not excluded that with the improvement of technology applied to molecular genetics, it will be possible to determine the PMI in a precise way, for example, although Next Generation Sequencing (NGS) is not yet widely employed in forensics, the use of NGS for necrobiome or thanatomicrobiome analysis will be an important research area in the near future, offering a potential alternative to classical PCR analysis, which complements the information collected from the cadavers, considering the necrobiome as a microbial clock indicator [64,65].

Currently, if we would identify practical indications for those who are interested in research in DNA for PMI estimation, considering the results reported by all the works reported in this review, we could suggest some guiding points:

- Whether a study on animal models or on human cadavers is chosen, make sure that the ethical standards of the research are respected;

- Begin to consider a study on human cadavers given that the rate of DNA degradation varies in samples from different organisms (animals or humans);

- Consider a sample group homogeneous by age range, gender and, if possible, previous pathologies;

- Corpses must have homogeneous characteristics, for example in terms of time since death, cause of death and environmental conservation conditions (temperature, humidity, pH values), avoiding highly degraded cadavers (burnt, skeletonized, exhumed);

- Choose only one tissue and make serial withdrawals over time. In particular, prefer brain for later PMI study and dental pulp or spleen/liver for early PMI study (considering that the rate of DNA degradation varies in different tissues of the same corpse);

- Chose an analysis method that is able to discriminate between human DNA and bacterial DNA, to avoid influence on the determination of the human PMI;

- Experimental conditions should be controlled in order to maximize consistency and accuracy;

- Keep in mind, in light of the previous points, to plan the research protocol relatively to the final purpose of studying early or later PMI.

We can conclude that in recent years several analytical techniques have been proposed to analyse the post-mortem DNA degradation in order to use it to estimate the PMI. Studies focused mainly on animal models and on particular tissues (spleen, heart, brain). The results have been mixed, with a prevalence of studies showing that the use of DNA degradation to estimate PMI is, to date, still unpromising. While on the one hand literature data in this field have confirmed knowledge already known in the forensic field, that is that in the post-mortem several degradation processes involve nucleic acids, on the other hand some fundamental aspects are still little explored: the influence of ante and post-mortem factors on DNA degradation, the feasibility and applicability of a multi-parametric mathematical model that takes into account DNA degradation and the definition of one or more target organs in order to standardize the results on human cases under standard conditions.

However, further studies could, thanks also to the use of innovative DNA extraction, amplification and quantification techniques, already used or recently updated for forensic purposes (such as RT-qPCR analysis), give more promising results, both in terms of application and statistical evaluation. 

## 4. Materials and Methods

This review has been performed following the Preferred Reporting Items for Systematic Reviews and Meta-Analyses and the PRISMA Guidelines [16].

The articles identified in the present review have been selected from PubMed, Google Scholar, Web of Science and Scopus databases; for the search strategy we decided to use the following keywords: “DNA [and] PMI” and “DNA degradation [and] PMI”. We used the keywords isolated or combined. We searched for more studies among the reference lists of the selected papers and systematic reviews. In this way, we identified a total of 487 works on the databases.

**Figure 2:**
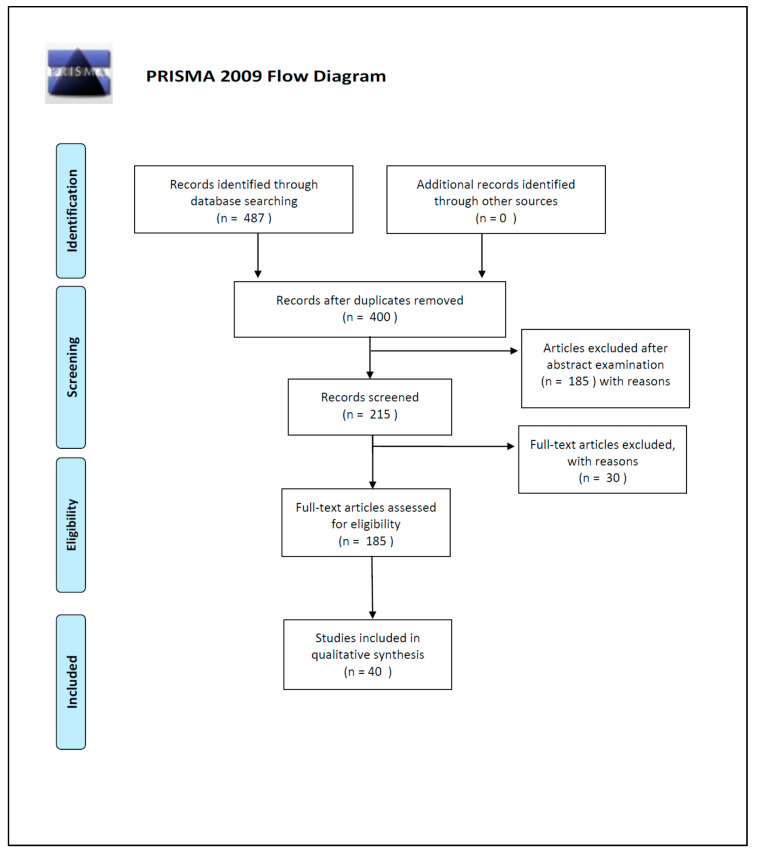
PRISMA 2009 Flow Diagram.

The initial search of the article was conducted by three reviewers (SS, PT and MS). They used the protocol of search previously described to identify literature. In case of disagreements, the consensus of the research supervisor (LC) was asked. The researchers used the following research order. Titles were screened first, then abstracts and full papers. A paper was considered potentially relevant and its full text reviewed if, following discussion between the two independent reviewers, it could not be unequivocally excluded on the basis of its title and abstract. The full text of all papers not excluded on the basis of abstract or title was evaluated. The number of articles excluded or included were registered and reported in a PRISMA flowchart (Figure 2).

Duplicates were removed and a total of 400 works’ abstracts were screened on the basis of the inclusion criteria: (1) abstract and/or full text in English language; (2) titles and/or abstracts suggested a PMI estimation related to DNA degradation rates; (3) titles and/or abstracts suggested the use of quantitative and/or qualitative DNA techniques used for PMI estimation. A total of 215 articles’ abstracts were selected during this phase of screening, after which 185 articles were examined in full text for eligibility. 

In the end, after full-text examination, we selected 40 experimental studies published between 1988 and 2019 for the qualitative synthesis. 

Data were extracted on: Author, Year of publication, Species used, Tissues and organs, Number of animals or subjects included in the study, Temperature (°C) of storage, Time frame assessed, Detection analytical methods, Statistical analysis used (if applicable) for PMI estimation, Limits of the study.

To estimate the potential bias that were most relevant for the study, we used the Cochrane tool for assessing risk of bias in randomized trials (RoB 2 tool) and the Risk of Bias In Non-randomized Studies of Interventions (ROBINS-I). All articles included have been considered of low risk bias.

## Figures and Tables

**Table 1:** Correlation between DNA degradation and post-mortem interval (PMI) at different points.

**Study**	Year	Species	Tissues amd Organs	Number	Temperature (°C)	Time Frame Assessed	Detection Methods	Statistical Analysis	Remarks
Perry et al. [17]	1988	Human	Rib bone	7	19–25	Weeks	Sothern blotting (RFLP ^a^)	-	The DNA degradation rate could vary with temperature and humidity more than it varies between individuals
Cina et al. [3]	1994	Human	Spleen	10	25	2 weeks	Flow cytometric analysis	-	Nuclear DNA was degraded in a relatively predictable fashion over time
Di Nunno et al. [4]	1998	Human	Spleen	35	2	24–126 h	Flow cytometric analysis	-	Constant relation between the time of death and DNA denaturation, particularly within the first 72 h after death
Lin et al. [18]	2000	Rat	Liver	15	-	0–24 h	Auto-TV image system	-	DNA degeneration rate of liver cells had a linear relationship to early PMI in rats
Liu et al. [19]	2001	Rat	Liver	15	-	0–48 h	Auto-TV image system	-	The degradation rate of DNA in nuclear cell had a certainty relationship to early PMI (in 48 h) of rat
Chen et al. [20]	2002	Human	Marrow in breastbone	1	20–25	7 d	Feulgen staining and computerized image analysis	-	The content of marrow DNA could be detected till 7 days after death
Di Nunno et al. [21]	2002	Human	Liver and spleen	25	-	24–65 h	Flow cytometric analysis	-	Hepatic tissue showed a virtually linear correlation between the time elapsed since death and the level of degradation of the DNA
Johnson et al. [22]	2002	Human	Blood cells	2	25	2, 22 h	Single-cell gel electrophoresis (SCGE)	Mean values of comet-tail-length and comet-tail-moment	The fragmentation of nuclear DNA increased with PMI in the 3–56 h post-mortem period
		Pig	Skeletal muscle, heart, liver and kidney	24	15	3–72 h			
Liu et al. [23]	2003	Rabbit	Cornea epithelial and endothelium	105	-	0–72 h	Computerized image analysis	-	The degradation rate of DNA in nuclear cell has an apparent relationship in 72 h after death of the rabbits
Boy et al. [7]	2003	Human	Teeth	57	-	24–144 h	Flow cytometric analysis	-	Dental pulp tissue exhibited minimal DNA degradation by 144 h post-extraction, and no constant relation was found between time and DNA degradation during this time
Liu et al. [24]	2004	Rat	Heart, liver, spleen and kidney	-	-	-	Flow cytometric analysis	-	The amount of DNA of all the viscera showed a decreased trend after death, especially in spleen
Long et al. [25]	2005	Human	Rib and teeth	-	15–20, 30–35	0–15 d	Flow cytometric analysis		DNA content of two kinds of tissue was degraded with the prolongation of PMI. There was a plateau period of 0–4 days for dental pulp cells of human being in 15–20 °C
Chen et al. [26]	2005	Human	Heart, liver, spleen and kidney	1	-	6–48 h	Feulgen staining and image analysis technique	-	The amount of DNA in human heart, liver and kidney had a more rapid degradation rate in first 6 h after death than in the spleen
Ren et al. [27]	2005	Human	Liver	18	-	4–36 h	Feulgen-Vans staining and computer image-analyze technique	-	DNA content declined regularly with the prolongation of time of death within 36 h
He et al. [28]	2005	Human	Spleen	-	4, 17–28	7–36 h	Feulgen staining and image analysis technique	-	The degradation rate of DNA had a certain relationship to early PMI (in 36 h)
Shu et al. [29]	2005	Human	Brain and spleen	32	16–25	5–36 h	Feulgen-Vans staining and image analysis technique	Linear regression analysis	DNA content declined regularly with the prolongation of PMI within 5–36 h
He et al. [30]	2005	Rat	Spleen	-	-	0–72 h	Single-cell gel electrophoresis (SCGE)	-	The fragmentation of nuclear DNA increased with PMI in the 0–15 h post-mortem period
He et al. [31]	2005	Rat	Liver	-	-	0–30 h	Single-cell gel electrophoresis (SCGE)	-	The fragmentation of nuclear DNA increased with PMI in the 0–18 h post-mortem period
Zhan et al. [32]	2005	Rat	Liver, kidney and spleen	-	20	0–48 h	Terminal deoxynucleotide transferase	-	The reminders of dUTP were decreasing along with the postmortem interval
Luo et al. [33]	2006	Human	Bone marrow	1	-	Up to 14 d	Computerized image analysis	-	The content of marrow DNA decreased gradually with prolongation of PMI, and could be detected till 14 days after death
Zhen et al. [34]	2006	Rat	Heart	111	-	0–72 h	Single-cell gel electrophoresis (SCGE)	-	DNA degradation of myocardium cells has a linear correlation with PMI up to 72 h
Ren et al. [35]	2007	Human	Thyroid	-	-	-	Image analysis technique		The average DNA content in the thyroid follicular epithelial continued to decrease with increased PMI
Chen et al. [36]	2007	Rat	Retina	90	20	0–28 h	Feulgen-Vans staining and image analysis technique	Linear regression analysis	In retinal nucleus, DNA content gradually declined with the prolongation of PMI
Liu et al. [8]	2007	Rat	Spleen	34	25	0–36 h	Feulgen staining and image analysis technique	Stepwise regression analysis	DNA content declined gradually within the first 36 h after death
El-Harouny et al. [37]	2008	Rat	Lung, liver, spleen, skeletal muscle and brain	40	-	0–24 h	Single-cell gel electrophoresis (SCGE)	Student’s t-test	Brain showed slower rate of DNA degradation than that of liver and lung
Hu et al. [38]	2008	Rat	Brain and bone marrow	-	1, 20	0–40 h	Single-cell gel electrophoresis (SCGE)	-	The linear relation between degradation of brain DNA and PMI was better than that of bone marrow
Fang et al. [39]	2010	Pig	Retina	-	15	2–24 h	Single-cell gel electrophoresis (SCGE)	Linear regression analysis	From 2 h to 24 h postmortem, the degree of degradation of retinal DNA increased with the prolongation of PMI
Zheng et al. [40]	2010	Rat	Skeletal muscle, heart, liver, kidney and brain	-	-	0–72 h	Single-cell gel electrophoresis (SCGE) + Auto-image analysis	Linear regression analysis	DNA content showed a decreasing tendency within 72 h post-mortem
Larkin et al. [41]	2010	Pig	Skeletal muscle	2	Summer and winter season	52, 81 d	PCR		A comparative seasonal analysis showed an overall decrease in DNA yield from 0 ADD to 101 ADD in summer and up to 138 ADD in winter
Xiong et al. [42]	2010	Rat	Kidney and liver	-	25	48–72 h	Raman micro-spectroscopy	-	DNA content in tissue cells decreased gradually with the prolongation of PMI from 48 to 72 h after death
Alaeddini et al. [43]	2011	Human	Rib bone	12	Room temperature and shallow burial	0–24 h	PCR	-	There was not a mathematical relationship between PMI and the level of degradation products in samples stored in the same environment
Nazir et al. [44]	2011	Rabbit	Skeletal muscle	60	-	0–7 d	PCR		DNA extracted from muscle taken from whole bodies gave 4-plex profiles on day 1 (13 ADD) and day 7 (112 ADD)
Itani et al. [45]	2011	Rat	Brain, liver, kidneys and skeletal muscle	-	4, 20	0–4 weeks 5–6 weeks	Real-time PCR	Student’s t-test	DNA decreased to below the value of 10 RFU in 1–3 weeks in the liver, kidney and skeletal muscle at 20 °C, while that in the brain was more than the value of 10 RFU for six weeks
Li et al. [46]	2011	Human	Liver	13	10, 20, 30	13–34 h	Feulgen staining and image analysis technique	Linear regression analysis	DNA degradation in liver cells showed linear correlation with PMI
Zheng et al. [9]	2012	Rat	Brain and teeth	111	22	0–72 h	Single-cell gel electrophoresis (SCGE)	Linear regression analysis	DNA degradation in brain and dental pulp cells showed linear relationship within 72 h after death
Gomaa et al. [47]	2013	Rat	Brain, liver and skeletal muscle	36	-	0–24 h	Single-cell gel electrophoresis (SCGE)	Pearson correlation	The brain and liver cells showed increased DNA degradation rate with prolongation of PMI within 24 h
Williams et al. [10]	2015	Human	Brain and spleen	15	4, 21	17–60 h	Flow cytometric analysis	Bonferroni correction	DNA degradation was more rapid in samples stored at room temperature as compared with refrigerated ones. Brain showed slower DNA decay than spleen
Ebuehi et al. [11]	2015	Rat	Brain, liver, heart and kidney	20	-	0–48 h	RAPD ^b^-PCR	-	DNA from brain, as compared to liver and kidney, showed a slower degradation rate
Zaki et al. [48]	2017	Rat	Brain and skeletal muscle	40	-	0–96 h	Single-cell gel electrophoresis (SCGE)	ANOVA ^c^	There was a good correlation between DNA fragmentation in brain and muscle tissue and PMI within 96 h after death
Mansour et al. [49]	2019	Human	Teeth	95 teeth from 39 corpses	Different temperature conditions	1 d–70 y	Real-time PCR		The highest DNA amount was observed in dental samples representing the shortest PMI. DNA concentration dropped substantially after 10 days after death. The early period after death is the most critical period with respect to yielding dental DNA

^a^ Restriction fragment length polimorphism, ^b^ Random Amplification of Polymorphic DNA, ^c^ Analysis of variance.

**Table 2:** Summary of the pros and cons of each of the techniques so far proposed in the literature for the estimation of PMI by DNA analysis.

Technique	Pros	Cons
**Restriction fragment length polimorphism analysis**	It has a historical value in the discipline since it is the first technique applied Limited costs	Very large fragments not suitable for evaluating DNA degradation Very few studies carried out to standardize method and experimental conditions Technique no longer used
**DNA flow cytometry analysis**	Quantitative technique, very sensitive Possibility to measure multiple characteristics of a cell	Difficulty in the forensic analysis of human samples without extensive manipulation Difficulty in differentiating human DNA from bacterial or fungal DNA Scarce encouraging results with some tissues
**Image Analysis Technique**	Various parameters can be acquired (ID, IOD, AOD, AG) Widely applied in animal models and human different tissues	It combines many techniques related to various disciplines High costs in terms of material and human resources Not specific because it doesn′t differentiate eukaryotic and prokaryotic DNA mixtures
**Single Cell Gel Electrophoresis**	Limited costs Quick technique Specific technique for quantifying DNA damage	Relatively insensitive and not very specific No standards available for verification Methodologies somewhat subjective Effectiveness dependant on the concentration of the DNA Laborious technique (analysis with many steps)
**DNA amplification analysis**	Highly specific and sensitive Widespread technique and reagents commonly available in molecular genetics laboratories Already used in forensic genetics A quantification technique can be used Quick technique Specific in differentiating eukaryotic and prokaryotic DNA mixtures	Relatively high costs Few systematic studies available so far in this field Target sequences must be known in advance

## Abbreviations

PMI	Post Mortem Interval
EPMI	Early Post Mortem Interval
IAT	Image Analysis Technique
SCGE	Single-cell Gel Electrophoresis
RT-qPCR	Real-Time quantitative Polymerase Chain Reaction
RFLP	Restriction Fragment Length Polymorphism
ID	Index of distortion
IOD	Integral Optical Density
AOD	Average Optical Density
AG	Average Grey
IA	Integral Absorbance
AA	Average Absorbance
SCGE	Single Cell Gel Electrophoresis
OTM	Olive Tail Moment
ADD	Accumulated Degree-Days
RFU	Relative Fluorescence Units
RAPD-PCR	Random Amplification of Polymorphic DNA- Polymerase Chain Reaction
TDT	Terminal Deoxynucleotidyl Transferase
dUTP	Deoxyuridine Triphosphate
dNTP	Deoxy-ribonucleoside Triphosphates
ROBINS-I	Risk of Bias In Non-randomized Studies of Interventions
NGS	Next Generation Sequencing
